# Roles and regulation of δ-catenin in tumorigenesis and neuronal diseases

**DOI:** 10.3389/fcell.2025.1559059

**Published:** 2025-03-27

**Authors:** Yang Zhang, Keping Xie, Tingting Jiang

**Affiliations:** Center for Pancreatic Cancer Research, The South China University of Technology School of Medicine, Guangzhou, China

**Keywords:** cancer, CTNND2, δ-catenin, neurodevelopment, neuro-diseases

## Abstract

*CTNND2* gene is located on the short arm of human chromosome 5 and encodes δ-catenin protein, which interacts with different proteins and plays different cell functions. Studies have demonstrated that δ-catenin plays an important role in regulating synaptic maturation and neuronal integrity. The *CTNND2* gene is closely associated with a variety of neurological diseases, including Cri-du-Chat syndrome, Autism spectrum disorders, Alzheimer’s disease, and Epilepsy. Furthermore, an increasing number of studies have demonstrated that *CTNND2* is involved in various cancers and may serve as a novel biomarker for the diagnosis and treatment for these diseases. In this review, we will focus on the signaling regulatory functions of *CTNND2* and its encoded protein δ-catenin in neuro-related diseases and cancers, and discuss the limitations of previous investigative studies and the challenges of the future researches on *CTNND2* and δ-catenin signaling.

## 1 Introduction

The *CTNND2* gene is situated on the short arm of human chromosome 5 and encodes a protein called delta-catenin/δ-catenin (referred as δ-catenin in this article) belonging to the armadillo/β-catenin superfamily (Paffenholz and Franke, 1997; Medina et al., 2000) and neural plakophilin-related armadillo repeat protein (NPRAP). The δ-catenin protein plays a variety of roles within cell, including the regulation of cell adhesion (Koutras and Lévesque, 2011; Koutras et al., 2011; Hatzfeld, 2005), neurodevelopment (Pauly et al., 2024; Tuncay et al., 2022; Wang L. Y. et al., 2024; Xu et al., 2023a; He et al., 2012; Vaz et al., 2023), and particularly formation and maintenance of dendritic spines and synapses (Yuan et al., 2015; Halder et al., 2015; Hassani et al., 2020; Adegbola et al., 2020; Wang S. et al., 2021; Li et al., 2021; Assendorp et al., 2024a; Ide et al., 1999; Izawa et al., 2002). Alterations in δ-catenin expression have been linked to the occurrence and progression of numerous pathological conditions, including autism (Zhang et al., 2016; Коваленко et al., 2020; Wang L. et al., 2024; Turner et al., 2015; Asadollahi et al., 2014; Lu et al., 2016; Zhou et al., 2019), schizophrenia (Chen et al., 2023; Vrijenhoek et al., 2008; Nivard et al., 2014), intellectual disability (Asadollahi et al., 2014; Einarsdottir et al., 2017; Hofmeister et al., 2015), and cancers (Oh et al., 2023; Wang et al., 2009; Burger et al., 2002; Huang et al., 2006; Zheng et al., 2004; Frattini et al., 2013).

In recent years, the study of δ-catenin has made remarkable progress due to the rapid development of molecular biology technology. It has been demonstrated that δ-catenin plays a crucial role in the regulation of synaptic maturation and neuronal integrity. The loss of function of δ-catenin may result in synaptic loss and neurodevelopmental disorders. Furthermore, the interaction between δ-catenin and the Wnt signaling pathway may play a significant role in tumor proliferation and drug resistance, offering a novel perspective for the treatment of cancers.

The aim of this article is to provide a timely review on the structure and function of *CTNND2* gene and its encoded protein δ-catenin, its relationship with human diseases, and the potential regulatory mechanisms in the physiological and pathological processes. Revealing the relationship between δ-catenin and diseases could provide a referable therapeutic direction for the treatment of related diseases.

## 2 Structure and function of *CTNND2* and δ-catenin

### 2.1 Structure and localization

**FIGURE 1:**
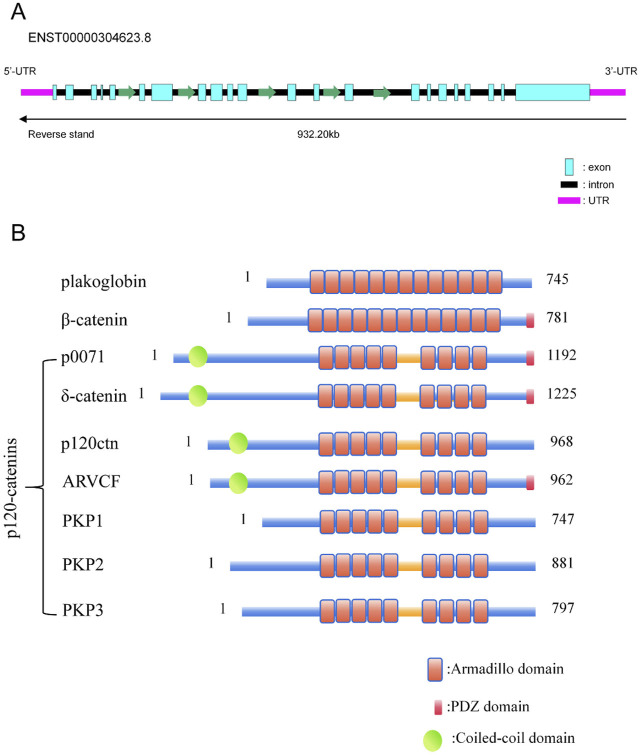
*CTNND2* Gene and the p120 protein family proteins. **(A)** Gene structure diagram of the transcript ENST00000304623.8 of the *CTNND2* gene. **(B)** Diagram of the structures of proteins with Armadillo and PDZ structural domains. The structure diagram of plakoglobin, β-catenin, and p120 catenins were summarized. All of the p120 protein family proteins, including p0071, δ-catenin, p120-catenin, ARVCF, PKP1, PKP2, and PKP3, contain a central armadillo structural domain, which consists of nine tandem incomplete 42-amino acid repeat sequences. p0071, Plakophilin-4 (PKP4); ARVCF, Armadillo Repeat Protein Deleted in Velo-Cardio-Facial Syndrome; PKP1, Plakophilin 1; PKP2, Plakophilin 2; PKP3, Plakophilin 3.

The *CTNND2* gene is situated on the short arm of human chromosome 5, within the 5p15.2 region, and spans approximately 1.2 Mb (10,971,836–11,904,446 bp). The nucleic acid structure of the *CTNND2* gene is shown schematically in Figure 1A, where one of the most widely studied transcripts contains 22 exons. Like all members of the subfamily of armadillo proteins (Keil et al., 2013; Peifer et al., 1992), δ-catenin protein possessed a central domain comprising a series of repeating motifs with approximately 40–45 amino acids in length, which is referred to as an arm repeat sequence (Paffenholz and Franke, 1997; Huang et al., 2021). The structure diagram is presented in Figure 1B. The arm repeat domain provides a multifunctional scaffold for protein-protein interactions, thereby enabling the participation in protein folding and stability, as well as interactions with other proteins (Huang et al., 2021). Consequently, some members of the armadillo protein-related protein subfamily, such as δ-catenin, can function in a variety of biological processes, including intracellular signaling, intercellular adhesion, and cytoskeletal organization. The C-terminal of δ-catenin protein contains a PDZ-binding motif, which contribute to its interaction with proteins containing PDZ domain, such as PSD95 and ABP/GRIP proteins. These interactions are critical for synaptic function and neurodevelopment (Yuan et al., 2015).

In normal human tissues, δ-catenin is predominantly expressed in the brain, with minimal expression observed in other normal human tissues. δ-catenin is predominantly localized to the cytoplasm (Lu, 2010; Wang et al., 2011) with potential nuclear localization capabilities (Koutras and Lévesque, 2011; Koutras et al., 2011), which may be associated with its involvement in transcriptional regulation. A comprehensive review of the existing literature is timely important to reveal a consistent association between the specific location of δ-catenin and a number of neurological disorders. The analysis of the protein structural domains specific to δ-catenin informs their participation and function in a variety of life activities.

### 2.2 Physiological functions

δ-catenin is a protein that plays a crucial role in cell adhesion (Koutras et al., 2011; Izawa et al., 2002), signal conduction (Bareiss et al., 2010; He et al., 2023; Dunbar et al., 2021), and neurodevelopment (Pauly et al., 2024; Hassani et al., 2020). δ-catenin can form a complex with other components of adhesion junctions and regulate adhesion molecules, including cadherin and β-catenin, during the process of cell adhesion, which further regulate the histomorphogenetic processes (Izawa et al., 2002; Bareiss et al., 2010; Lu et al., 1999; Ligon et al., 2020; Kim et al., 2002). In addition, δ-catenin has been identified as a nervous system adhesion protein that undergoes dynamic reorientation during neurodevelopment (Ho et al., 2000). δ-catenin proteins can be modified by different post-translational modifications, including phosphorylation (Li et al., 2021; Zhou et al., 2019; Bareiss et al., 2010; Chen et al., 2021; Qu et al., 2024), methylation (Arpón et al., 2019; Singh et al., 2015), and palmitoylation (Zhang X. L. et al., 2018), during the process of cell signaling. Therefore, the localization, structure, interaction, and function of δ-catenin can be changed after these modifications.

δ-catenin plays a significant role in neurodevelopment by regulating the maturation of dendritic spines, synaptic maintenance, and neuronal excitability. Assendorp *et al.* have shown that δ-catenin can impede the maturation of synaptic connections and simultaneously promote the integrity of neuronal cells. In addition, δ-catenin can regulate neuronal excitability during postnatal development; and maintain synaptic integrity in adulthood. δ-catenin plays a crucial role in regulating neuronal excitability and synaptic plasticity (Assendorp et al., 2024b). Deficiency of *CTNND2* may lead to impairment of synaptic function. For example, the knockout of *CTNND2* gene induces sleep wake disorders in mice (Xu et al., 2023b). *CTNND2*-KO mice exhibit typical autism-like behaviors, suggesting that *CTNND2* gene is important in the processes of spatial learning and memory, as well as in the mechanisms of Rictor-mediated actin polymerization and synaptic plasticity (Wang X. et al., 2021). Another study demonstrated that *CTNND2*-KO mouse models exhibit behaviors consistent with autism spectrum disorder (ASD) and exhibit decreased dendritic spine density in the hippocampus. Melatonin can improve the synaptic function of gamma-aminobutyric neurons by activating the PI3K/Akt signaling pathway, which may be related to the improvement of social behavior deficits and dendritic spine damage in *CTNND2*-KO mice (Wang L. Y. et al., 2024). These studies provide possible therapeutic options for disorders related to neurodevelopmental defects, such as autism caused by *CTNND2* deletion.

Additionally, δ-catenin may play a role in the evolution of the human brain. δ-catenin may promote synaptic regeneration through the regulation of SRGAP2C in human evolution, suggesting that δ-catenin may promote the development and function of the human brain by regulating the maturation rate of synapses (Assendorp et al., 2024b). δ-catenin also plays a role in spatial learning and memory through rictor-mediated actin aggregation and synaptic plasticity (Wang S. et al., 2021). Moreover, abnormal δ-catenin expression or function has been linked to a range of neurological disorders, including intellectual disabilities (Hofmeister et al., 2015), autism spectrum disorder (Tuncay et al., 2022; Wang L. et al., 2024; Turner et al., 2015; Xu et al., 2023b), and schizophrenia (Chen et al., 2023; Vrijenhoek et al., 2008). The following section will address the relationship between δ-catenin and neurosystem-related diseases.

## 3 δ-catenin in diseases

The *CTNND2* gene is located in close proximity to the lesions of the neurodevelopmentally related disease Cri-du-Chat syndrome. Alterations in *CTNND2* gene are strongly associated with severe mental retardation in Cri-du-Chat syndrome. Furthermore, recent studies have substantiated the correlation between *CTNND2* and other neurological disorders, including Epilepsy, Autism, Alzheimer’s disease, and Myopia. An increasing number of studies have indicated that δ-catenin may play an important role in the progression of multiple types of cancer. Therefore, we shall present an overview on the existing studies related to *CTNND2*, outlining the relationship between *CTNND2* gene and certain types of diseases and providing suggestions on the treatment of related diseases according to the different changes of *CTNND2* in different diseases.

### 3.1 *CTNND2* and neurological diseases

#### 3.1.1 *CTNND2* and cri-du-chat syndrome

Cri-du-Chat syndrome is a chromosomal deletion syndrome, in which a portion of chromosome 5 is absent (Chhaya and Chan, 2013; Almeida et al., 2023). The deletion typically encompasses the 5p15.2–5p15.3 region, where the *CTNND2* gene is situated (Sardina et al., 2014). In fact, the deletion of the *CTNND2* gene is linked to a range of abnormalities, including facial deformities, microcephaly, cerebellar hypoplasia, lateral ventricle enlargement and severe psychomotor development delays, which are hallmarks of Cri-du-Chat syndrome (Sheth et al., 2012). In Cri-du-Chat syndrome, deletion or abnormal function of the *CTNND2* gene can lead to severe intellectual impairment and developmental delays in the nervous system (Medina et al., 2000). In Cri-du-Chat syndrome, the deletion of the *CTNND2* gene not only affects the development of the nervous system, but may also impact the development of other organs, resulting in a complex set of clinical symptoms. For example, in one clinical case, a full-term female infant presented with ischemic retina and retinal hemorrhage (Chhaya and Chan, 2013). Therefore, the function of the δ-catenin in Cri-du-Chat syndrome is multifaceted, including specific neuronal proteins that affect brain development, which in turn affect normal brain development, and its deletion ultimately results in developmental delays in the nervous system.

#### 3.1.2 *CTNND2* and autism spectrum disorder

The relationship between the *CTNND2* gene and autism spectrum disorder is gradually appreciated. δ-catenin plays an important role in the development of the nervous system, particularly in the formation and functionality of synapses (Wang L. Y. et al., 2024). The *CTNND2* gene is regarded as a novel autism gene and its encoded δ-catenin protein is a neuron-specific protein and plays a role in cell adhesion and dendritic branching. Abnormal dendritic spines have been observed in the cerebral cortex of both patients and mouse models of autism spectrum disorder, which is related to the dysfunction of the *CTNND2* gene (Wang L. et al., 2024; Ligon et al., 2020). The *CTNND2* gene knockout mice exhibited such symptoms as social behavior disorder and decreased dendrite spine density, which may be related to an imbalance of excitatory and inhibitory neurotransmitters (Wang L. Y. et al., 2024; Xu et al., 2023a; Wang X. et al., 2021). Moreover, some studies have corroborated the existence of tandem duplication (Miller et al., 2020), missense, and dose sequence variation (Tuncay et al., 2022; Turner et al., 2015) in the genes of patients with autism, behavioral problems and malformation characteristics, indicating that anomalous *CTNND2* level may be associated with the severity of autism and the cognitive phenotype. Therefore, normal *CTNND2* gene and its δ-catenin expression are critical for neurodevelopment, and aberrant δ-catenin expression is evident in patients with Autism Spectrum Disorder.

#### 3.1.3 *CTNND2* and myopia

Alterations in δ-catenin expression, predominantly in the nervous system of the brain, are associated with a range of neurological disorders. For instance, *CTNND2* has been implicated in the development of myopia. Adegbola et al. (2020) found that the destruction of the *CTNND2* gene is correlated with the development of attention deficit hyperactivity disorder and myopia, through genetic analysis in several members belongs to a family. Yu *et al.* observed significant differences in the distribution of variants of SNP rs1479617, located within the *CTNND2* gene, between the pathological and control groups (Yu et al., 2012). Polymorphisms in the specific *CTNND2* gene and 11q24.1 genomic region were significantly associated with pathological myopia in the Chinese population (Yu et al., 2012). Lu *et al.* demonstrated a robust correlation between *CTNND2* polymorphism and myopia using Sanger sequencing (Lu et al., 2011). Li *et al.* found a robust association between *CTNND2* and high myopia in an Asian cohort (Li et al., 2011b). Wen *et al.* demonstrated that extrachromosomal circular DNA (eccDNA) levels of *CTNND2* were significantly increased in the anterior lens capsule of highly myopic patients (Wen et al., 2023). Liu *et al.* conducted a comprehensive meta-analysis of myopic patients and controls, and revealed a significant association between myopia and two specific genetic variants, rs6885224 and rs12716080, of the *CTNND2* gene (Liu and Zhang, 2014). In addition, some researchers have also shown a strong correlation between *CTNND2* and high myopia through animal studies (Yin et al., 2019; Srinivasalu et al., 2018) and data from clinical studies in different regions (Liu J. et al., 2021; Li et al., 2011a). These findings suggested that the *CTNND2* gene may play an important role in the development of high myopia.

#### 3.1.4 *CTNND2* and other neurological diseases

*CTNND2* is one of the genes that regulate the development of brain neurons. Four single-nucleotide polymorphisms (SNPs) of the *CTNND2* gene have been identified as being associated with schizophrenia (SZ), indicating that *CTNND2* gene be involved in the susceptibility to SZ (Chen et al., 2023), and *CTNND2* and some other genes important in neuronal function were disrupted in schizophrenia patients (Vrijenhoek et al., 2008). *CTNND2* has been identified as the causative gene of Dutch family corticoclonic tremor and epilepsy, since *CTNND2* missense mutations have been found in samples from patients with familial cortical myoclonic tremor and epilepsy (van Rootselaar et al., 2017).

Additionally, genetic variations in the *CTNND2* gene may contribute to the development of cortical cataracts of midlife patients and the subsequent structural and functional changes in the brain of Alzheimer’s disease (AD) patients (Jun et al., 2012). A rare missense mutation (G810R) located within the *CTNND2* gene alters the intracellular distribution of δ-catenin and increase secretion of amyloid beta protein (Aβ_1-42_) in nerve cell cultures, while amyloid-β (Aβ) is a neuropathological biomarker of AD (Jun et al., 2012). Moreover, Moncaster *et al.* have also proposed a potential association between *CTNND2* and AD (Moncaster et al., 2022). A review of the extant literature reveals a correlation between δ-catenin expression malfunction and certain psychiatric disorders. This correlation may be attributable to the location of and the unique structural domain of the δ-catenin protein. Further research on δ-catenin could offer novel insights into the treatment of neurological diseases.

#### 3.1.5 *CTNND2* variants in diseases

With the development of sequencing technology, there is increasing evidence that variants in the *CTNND2* gene are strongly associated with a number of diseases. Kang *et al.* applied whole exon sequencing to identify variants in the *CTNND2* gene, and discovered that these variants were linked to an increased risk of early-onset depression (Kang et al., 2021). Belcaro *et al.* reported two cases of internal deletion of the *CTNND2* gene detected by molecular karyotyping in patients who presented with isolated intellectual disabilities clinically, and pointed out that the deletion of the *CTNND2* gene was associated with intellectual disability (Belcaro et al., 2015). Hofmeister *et al.* combining human genetic and *in vivo* data with zebrafish embryos, found that impaired neuronal migration resulting from insufficient doses of the *CTNND2* gene may underlie cognitive dysfunction in patients with borderline intelligence and learning problems within the dyslexia spectrum (Hofmeister et al., 2015). Medina *et al.* found a strong correlation between haploid deletion of the *CTNND2* gene and the severity of intellectual disability, which suggests that when only one copy of the *CTNND2* gene is present, it plays an important role in the intellectual impairment of Cri-du-Chat syndrome (Medina et al., 2000). Therefore, the aberrant expression of δ-catenin is frequently observed in numerous neurologic diseases, indicating a potential correlation between δ-catenin and disease pathophysiology.

### 3.2 *CTNND2* and cancer

#### 3.2.1 *CTNND2* and neuro-associated cancers

Cancer is a leading cause of death worldwide due to the abnormal proliferation and structural heterogeneity of tumors, as well as the high tendency to metastasize. Earlier studies have shown that δ-catenin is involved in neurodevelopment. However, an increasing number of studies have demonstrated that δ-catenin plays a crucial role in the progression of neuro-related cancers. For example, Wang *et al.* identified the presence of δ-catenin protein in the cytoplasm of astrocytoma cells, and established a correlation between δ-catenin expression and the malignant progression of astrocytoma (Wang et al., 2011). Furthermore, they demonstrated that δ-catenin promotes the invasion of astrocytoma cells by upregulating Rac1 activity. Frattini *et al.* demonstrates that loss-of-function mutations in *CTNND2* target neurospecific genes and are associated with the transformation of glioma cells along a highly aggressive mesenchymal phenotype (Frattini et al., 2013). Shimizu *et al.* demonstrated that overexpression of δ-catenin increased the *ex vivo* invasiveness of glioma cells, whereas knockdown of δ-catenin decreased the invasiveness of glioma cells, suggesting δ-catenin as a potential therapeutic target for the treatment of aggressive glioma when used in combination with bevacizumab (Shimizu et al., 2019). Hu *et al.* demonstrated that the expression level of δ-catenin in tumor tissues of medulloblastoma patients was significantly elevated in comparison to normal tissues (Hu et al., 2022). This may potentially impede the invasion of medulloblastoma cells by inhibiting the epithelial-mesenchymal transformation (EMT) pathway, and may also predict a favorable prognosis of medulloblastoma patients. Collectively, the aberrant expression of δ-catenin is frequently observed in neural-related cancers. However, the current research on δ-catenin in nerve-related cancers is limited and underlying mechanism remains to be elucidated. The more studies are needed to explore the clinical significance of δ-catenin as a biomarker for diagnosis and treatment of those cancers.

#### 3.2.2 *CTNND2* and other cancers

In addition to nerve-related cancers, aberrant expression of δ-catenin is also seen in other cancers. Huang et al. (2018) demonstrated that the removal of the *CTNND2* gene resulted in a notable reduction in the tumorigenesis and metastatic ability of Lewis lung cancer cells. Conversely, the overexpression of δ-catenin enhanced the subcutaneous tumorigenesis and distant. The expression levels of a group of linking genes, including *CTNND2*, were significantly correlated with the overall survival rate of lung adenocarcinoma patients (Xie et al., 2024). Wang *et al.* demonstrated that δ-catenin (*CTNND2*) may serve as a prognostic biomarker for lung adenocarcinoma (Wang et al., 2020). Therefore, δ-catenin may play an important role in the pathogenesis of lung adenocarcinoma.

In human prostate cancer, δ-catenin displays exon mutations and promotes cancer cell survival, adaptation and metabolic reprogramming (Nopparat et al., 2015). Nucleotide polymorphisms in the 5′untranslated region (UTR) of the *CTNND2* gene were linked to a high Gleason score and poorly differentiated prostate adenocarcinoma (Wang et al., 2009). Furthermore, functional nonsense mutations in *CTNND2* have been identified as a promotor in the development of prostate cancer. The rearrangement of *CTNND2* loci, including gene replication, is a prevalent phenomenon in clinically significant prostate cancer (Zhang P. et al., 2018). This may serve as a potential underlying mechanism for δ-catenin overexpression. Lu *et al.* identified a coordinated regulation of δ-catenin expression by E2F1 and Hes1 in prostate cancer progression (Lu et al., 2010). Both the activating transcription factor E2F1 and repressive transcription factor Hes1 regulated the expression of δ-catenin (Lu et al., 2010). Other studies have found that the expression levels of PSMA and δ-catenin in prostate cancer tissue are significantly higher than those in normal prostate tissue, pointing that these two proteins could serve as potential diagnostic biomarker for prostate cancer (Burger et al., 2002).

In liver cancer, a number of studies have proven that δ-catenin may play a role in the progression of liver cancer. Hypoxia can induce the expression of δ-catenin, thereby promoting hepatocellular carcinoma (HCC) progression through the Wnt signaling pathway (Huang et al., 2019). Hypoxia can also induce the expression of EIF3J-AS1 and δ-catenin, and result in the downregulation of miR-122-5p in HCC cells and enhanced proliferation, migration, and invasion of HCC cells (Yang et al., 2019). Zhu *et al.* identified 10 genes associated with iron -dependent cell death, including δ-catenin (Zhu et al., 2022). Yue *et al.* proposed that δ-catenin may represent a promising therapeutic target for the treatment of intrahepatic cholangiocarcinom (Yue et al., 2023). Additionally, recent studies have indicated that δ-catenin may play a role in the progression of esophageal cancer and may serve as a potential biomarker for esophageal cancer through single-cell RNA sequencing analysis from esophageal adenocarcinoma patients and matched normal-adjacent tissue (Maity et al., 2022). Singh *et al.* employed genome-wide methylation analysis to ascertain that *CTNND2* promoter was hypomethylated and overexpressed in esophageal cancer (Singh et al., 2015). A combination of bioinformatics analysis and experimental verification has demonstrated that δ-catenin is significantly correlated with the prognosis of patients with pancreatic ductal adenocarcinoma (Zhang Y. L. et al., 2018). In breast cancer, δ-catenin is a potential contributor to the progression of breast cancer. δ-catenin may be a direct target of HOXB7, which is overexpressed in breast cancer (Heinonen et al., 2015).

Malignant transformation is frequently accompanied by loss of cell polarity and changes in cell morphology and the alterations in the connections between cancer cells and neighboring cells. δ-catenin is a cell junction protein and its change in expression is likely involved in many processes of cancer cell changes. δ-catenin as a biomarker for early diagnosis of cancers deserves further and in-depth investigation.

### 3.3 *CTNND2* and other diseases

Most of the existing literature on δ-catenin has been focused on neurological related diseases and cancer. However, δ-catenin is not only closely associated with neuro-related diseases and cancers, but also associated with inflammation, pain, kidney disease, diarrhea, diabetes, dental health, stroke, and so on. First, δ-catenin is upregulated in patients with asthma, and δ-catenin is the target of miR-218-5p. The mum-mir-218-5p expression was also decreased with δ-catenin expression increased in the airway of mouse model of allergic airway inflammation (Liang et al., 2020). Furthermore, δ-catenin is markedly abundant in cadherin-mediated pathways that regulate inflammation of cerebrovascular endothelial (Mackinnon et al., 2016).

Second, pancreatitis patients, especially chronic pancreatitis and recurrent acute pancreatitis, suffer different degrees of pain and persistent inflammation. δ-catenin was associated with all forms of pain and all causes of pancreatitis, and three SNPs of *CTNND2* gene was linked to the pain of pancreatitis by using candidate genetic association investigation (Dunbar et al., 2021). The gene region of *CTNND2* is associated with postoperative analgesic orthognathic surgery (Nishizawa et al., 2022).

Third, the δ-catenin expression level was higher in kidney tissue from scleroderma renal crisis patients by immunostaining analysis (Stern et al., 2020). δ-catenin is a potential biomarker that contribute to the formation of small-vessel stroke (Cárcel-Márquez et al., 2024). The δ-catenin level from peripheral blood may be potential diagnostic biomarker for ischemic strokes (Simats et al., 2020).

Forth, Nrf2 can regulate Wnt signaling by targeting δ-catenin, which explains the negative effects of gestational diabetes on lung development in late embryonic development and may be used clinically to prevent and treat lung developmental abnormalities caused by gestational diabetes (He et al., 2023). Besides, δ-catenin may be a biomarker for insulin-related disease risk (Arpón et al., 2019).

Finally, δ-catenin is associated with malaria (Maity et al., 2022), dental health (Lauritano et al., 2019; Ma et al., 2015), placental abruption (Workalemahu et al., 2018), buffalo milk production (Erdoğan et al., 2024; Du et al., 2019), and pig farming (Liu H. et al., 2021; Zhao et al., 2022). Thus, it is clear that δ-catenin, which regulates cellular adhesion and nerves, is involved in a number of biological processes, and is not only associated with disease, but also has the ability to influence many aspects of biological traits. A comprehensive summary of δ-catenin functional studies is presented in Table 1.

**TABLE 1:** Integrated Analysis of Functional Studies on δ-catenin.

Research dimension	Experimental model	Key findings	Technical approach	Comments	References
Molecular mechanism	Mouse model of Nrf2 deficiency	Nrf2 can regulate Wnt signaling by targeting *CTNND2*	CRISPR/Cas9 knockout + RNA-seq	The in-depth study of the specific molecular mechanism is not comprehensive enough	He et al. (2023)
	Prostate cell lines	δ-catenin forms complexes with E-cadherin, p120, α-catenin, β-catenin in PCa cells	Western blot + Immunoprecipitation	δ-catenin forms complexes with E-cadherin, p120, α-catenin, β-catenin in PCa cells	Zhang et al. (2018b)
	C57BL/6 and ICR mice	HIF1α can directly upregulate *CTNND2* gene expression	CRISPR/Cas9 knockout	There is no molecular evidence for the interaction between HIF and δ-catenin	Huang et al. (2019)
Epigenetic regulation	HEK293T cells	bFGF phosphorylates tyrosine residues of δ-catenin in Rv-1 cells	Western blot	The phosphorylation site of delta-catenin was not completely identified. The regulatory mechanism of delta-catenin stability is not well studied	Chen et al. (2021)
	HEK293T cells and C57BL/6J mice	δ-catenin is transiently palmitoylated by DHHC5 following enhanced synaptic activity	Western blot + Co-IP + Palmitoylation Assay	Lack of validation in animal experiments; The palmitoylation detection method has limitations	Brigidi et al. (2014)
	Human lung adenocarcinoma cell line A549	δ-catenin can regulate MTA2 in a methylation-dependent manner *via* Kaiso	Methylation specific PCR and ChIP experiments	The sample size is relatively small; Lack of animal experiments to simulate the environment *in vivo*	Dai et al. (2011)
Protein interaction network	SRGAP2 mouse strain and HEK293T cells and Human ESC H9 cells	*CTNND2* and SRGAP2 work together to regulate maturation, which may be related to human evolution	Co-IP + CRISPR/Cas9 gene knockout + Immunofluorescence	Focusing only on cortical areas of the brain, these findings may not generalize to other areas of the brain	Assendorp et al. (2024a)
Subcellular localization	U251 and U87 cell lines	δ-catenin expression was detected in the cytoplasm of astrocytoma cells	Immunohistochemistry	There is also literature suggesting that δ−catenin is expressed in the nucleus of other tissues or cells	Wang et al. (2011)

## 4 Regulatory mechanisms of δ-catenin

δ-catenin also participates in the Wnt/β-catenin signaling pathway and further affect cell function. Wnt/β-catenin signaling pathway is frequently associated with the onset and progression of tumors. Notably, δ-catenin has a variety of post-translational modifications, including phosphorylation (Luckert et al., 2011), methylation (Arpón et al., 2019), and palmitoylation (Zhang X. L. et al., 2018), which can alter the localization, structure, interaction, and function of the protein. Moreover, the prior research on δ-catenin focuses on neurodevelopment primarily and synaptic maturation and differentiation. The following section will discuss the epigenetic modification processes associated with *CTNND2* and the relevant signaling mechanisms.

### 4.1 Epigenetic regulatory mechanisms of δ-catenin

#### 4.1.1 δ-catenin and phosphorylation

In the prostate cancer cells, the tyrosine residues of δ-catenin can be phosphorylated after basic fibroblast growth factor (bFGF) interacting with fibroblast growth factor receptor 1 (FGFR1) (Chen et al., 2021). After phosphorylation, the binding between δ-catenin and glycogen synthase kinase 3β (GSK3β) was weakened, while the stability of δ-catenin was enhanced, facilitating the nuclear transport of β-catenin. δ-catenin phosphorylation promoted E-cadherin process and increased the total protein expression level, and result in the nuclear redistribution of β-catenin, thus enhancing the proliferation and migration of prostate cancer cells. Furthermore, the function of δ-catenin in the Wnt/β-catenin signaling pathway can also be regulated by phosphorylation. Phosphorylated δ-catenin enhanced the intracellular transport of β-catenin and the transcriptional activity of the downstream genes, by interacting with β-catenin. δ-catenin phosphorylation can also be mediated by other kinases, including Src family kinases, focal adhesion kinase (FAK), Janus kinase (JAK) (Chen et al., 2021), and MAPK JNK (Edbauer et al., 2009). The Ser-447 in δ-catenin is phosphorylated by MAPK JNK in a synaptic activity-dependent manner in neurons (Edbauer et al., 2009). A δ-catenin mutant defective in Ser-447 phosphorylation showed enhanced ability to promote dendrite branching in cultured neurons (Edbauer et al., 2009).

#### 4.1.2 δ-catenin and methylation

δ-catenin can regulate the expression of MTA2, D1 and MMP7 in a methylation-dependent manner via Kaiso, a δ-catenin-bound transcription factor. Methylation-specific PCR and ChIP analysis revealed that δ-catenin regulated gene expression via Kaiso and DNA methylation, e.g., regulation of MTA2 expression by δ-catenin through Kaiso depends on the methylation status of the MTA2 promoter (Dai et al., 2011). In addition to DNA and protein methylation, the methylation of *CTNND2* mRNA may play a significant role in regulating its expression and function, given that m^6^A methylation is a prevalent RNA modification that can influence mRNA stability, translation efficiency, and localization. *CTNND2* methylation and δ-catenin interactions with other proteins involved in methylation are also important in signaling and gene regulation, including Wnt/β-catenin signaling (Vershinin et al., 2016) and Wnt signaling (Lu et al., 2015).

#### 4.1.3 δ-catenin and palmitoylation

The palmitoylation of δ-catenin is mainly regulated by DHHC5, a palmitoyl transferase. DHHC5 enhances the hydrophobicity of δ-catenin by conjugating palmitic acid to the cysteine residue of δ-catenin, thereby promoting its localization and function on the cell membrane. Following enhanced synaptic activity, the cadherin-binding protein δ-catenin is subjected to a brief palmitoylation process by DHHC5, which enhances the interaction between δ-catenin and cadherin at the synapse (Brigidi et al., 2014). The palmitoylation of δ-catenin plays a crucial role in synaptic plasticity and memory formation. Increased synaptic activity induces palmitoylation of δ-catenin, a process that increases the interaction of δ-catenin with N-cadherin, thereby stabilizing synaptic connections and promoting extension of the postsynaptic spine. Specifically, palmitoylated δ-catenin contributes to the stabilization of N-cadherin in the synapse and promotes the insertion of GluA1 and GluA2 subunits into the synaptic membrane, thereby increasing the amplitude of the small excitatory postsynaptic currents (Brigidi et al., 2014). Furthermore, palmitoylation of δ-catenin has been demonstrated to be a critical factor in the pathogenesis of neuropathic pain. In a rat model of neuropathic pain, there was an increase in the levels of palmitoylated delta-catenin and palmitoyltransferase DHHC3 in sensory neurons of the dorsal root ganglion (DRG). Inhibiting palmitoyl transferase or reducing the abundance of delta-catenin in DRG may alleviate neuropathic pain caused by oxaliplatin or nerve damage. The palmitoylation of δ-catenin induced by the inflammatory cytokine TNF-α promotes its interaction with the voltage-gated sodium channel Nav1.6 and the driver protein KIF3A. This interaction facilitates the transport of Nav1.6 across the plasma membrane of DRG neurons, which in turn leads to mechanical hypersensitivity and abnormal pain (Zhang X. L. et al., 2018)

### 4.2 Wnt/β-catenin signaling pathway

The Wnt/β-catenin signaling pathway plays a crucial role in a multitude of biological processes, including cell proliferation, migration, invasion, EMT, angiogenesis, immune escape, phenotypic transition, clonal evolution and drug resistance (Ramakrishna et al., 2023). As a regulator of the Wnt/β-catenin signaling pathway, the abnormal expression and function of δ-catenin in cancers may be closely related to cancer development and progression. δ-catenin may enhance survival and adaptation of cancer cells by promoting activation of the Wnt/β-catenin signaling pathway and metabolic reprogramming, thereby supporting the growth and progression of tumor.

δ-catenin may play a significant role in the malignant progression through the activation of canonical Wnt signaling and the maintenance of cancer stem cells (Huang et al., 2018). In prostate cancer cells, δ-catenin is present in complexes with E-cadherin, p120 and α-catenin and β-catenin. Increased expression level in δ-catenin results in its further stabilization, as well as the stabilization and upregulation of its binding partners, including E-cadherin, p120, α-catenin and β-catenin. Anti-degradation and overexpression of δ-catenin isomers activate the Wnt signaling pathway by increasing nuclear β-catenin levels and subsequently stimulating Tcf/Lef transcriptional targets (Zhang P. et al., 2018). Hypoxia induced δ-catenin to promote the progression of liver cancer in mice through the Wnt signaling pathway. The hypoxia-induced transcription factor HIF1α can directly upregulate the expression of δ-catenin. The increased expression of δ-catenin then leads to an increase in its binding partner, which further leads to an increase in the expression of β-catenin, and leads to activation of the Wnt signaling pathway (Huang et al., 2019). Furthermore, nuclear factor erythroid 2-related factor 2 regulate the Wnt signaling pathway by targeting δ-catenin by RNA sequencing and luciferase reporter gene analysis (He et al., 2023).

**FIGURE 2:**
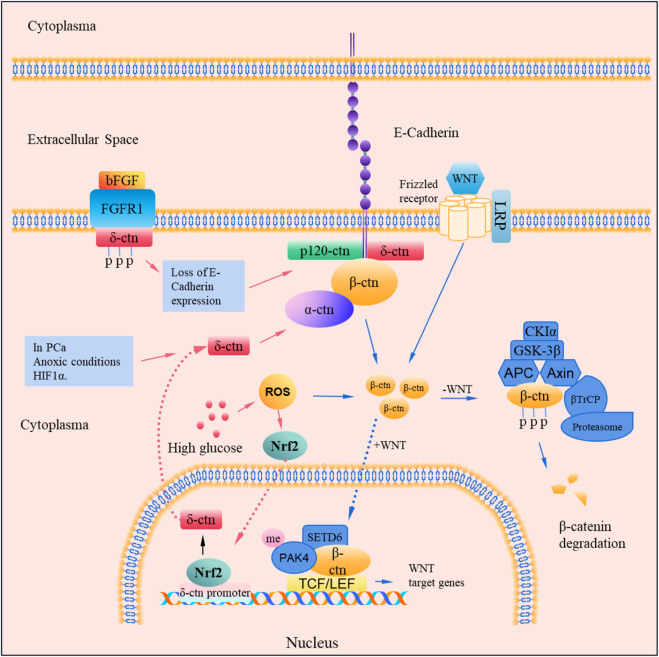
Mechanisms of *CTNND2* involved in the WNT/β-catenin pathway. δ-catenin exists in protein complexes with E-cadherin, p120, α-catenin and β-catenin. Increased expression of δ-catenin leads to upregulation and stabilization of its binding partners and can activate the Wnt signaling pathway by increasing the nuclear β-catenin level which in turn can stimulate the activation of TCF/LEF transcriptional targets. In prostate cancer cells, bFGF can phosphorylate the tyrosine residues of δ-catenin mediated by FGFR1, and enhance fragmentation of E-Cadherin, and promote intranuclear redistribution of β-catenin. Nrf2 increases the expression of *CTNND2*, which regulates the WNT/β-catenin signaling pathway. In the WNT signaling pathway, β-catenin stabilizes and binds to TCF/LEF in the nucleus to regulate target genes; and β-catenin can be phosphorylated and degraded by the GSK-3β protein complex.

In addition, studies have found that *CTNND2* gene mutation can also participate in the Wnt/β-catenin signaling pathway. Functional nonsense mutations in *CTNND2* promote the development of prostate cancer, and these mutations affect the activity of the Wnt/β-catenin signaling pathway. This suggests that δ-catenin may contribute to the progression of prostate cancer by affecting the Wnt/β-catenin signaling pathway. The mechanisms underlying δ-catenin-mediated regulation of WNT/β-catenin signaling pathway are summarized in Figure 2.

### 4.3 Other signaling pathways

In addition to its involvement in the Wnt/β-catenin pathway, δ-catenin regulates neurodevelopment and signaling *in vivo* through other pathways. For example, δ-catenin can slow synaptic maturation and promote neuronal integrity; human specific protein SRGAP2C can enhance synaptic accumulation of δ-catenin in human neurons. Besides, *CTNND2* deficiency can lead to loss of SYNGAP1 synapse. δ-catenin sets the pace of synaptic maturation and contributes to synaptic regeneration of human neurons through regulation of the human-specific protein SRGAP2C. δ-catenin is essential for the synaptic accumulation of SYNGAP1 and the formation of ID/Autism Spectrum Disorder-related protein complexes, which are uniquely regulated in humans and shape the synaptic developmental trajectories (Assendorp et al., 2024b).

During the differentiation of primary neurons, δ-catenin levels are increased while REST and TRIM28 protein levels are decreased, and the δ-catenin expression may be co-regulated by REST and TRIM28 protein (Lee et al., 2016). Moreover, δ-catenin binds to the last PDZ domain of S-SCAM through its carboxyl terminal, suggesting that δ-catenin regulate the molecular organization associated with synaptic connection through its interaction with S-SCAM (Ide et al., 1999). δ-catenin is also a potential iron death-related gene and subject to post-transcriptional regulation by circRNA/miRNA and m1A/m5C/m6A modifications in HCC (Zhu et al., 2022).

**FIGURE 3:**
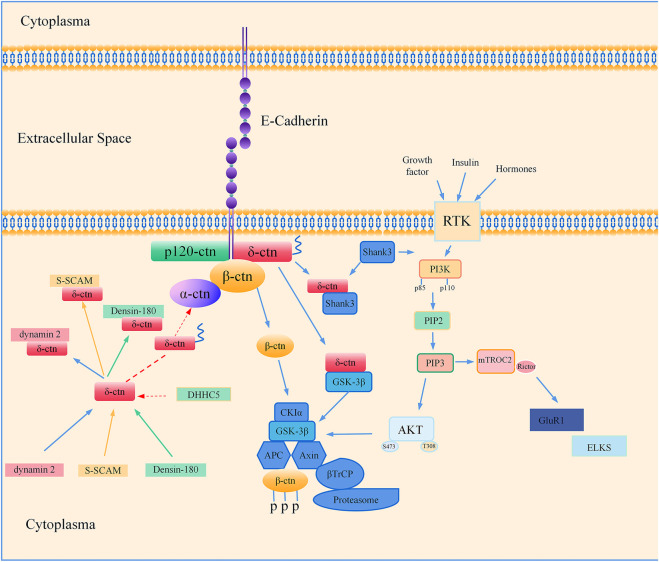
Regulatory mechanisms of δ-catenin associated with neural development and function. Co-localization and direct interaction of dynamin 2/NPRAP in neuroblastoma *in vivo*; NPRAP/δ-catenin is involved in synaptic junctions through its carboxy-terminal binding to the last PDZ structural domain of S-SCAM; Densin-180 co-localizes with δ-catenin/NPRAP; δ-catenin is transiently palmitoylated by DHHC5, and palmitoylation increases δ-catenin-adhesion protein interactions at the synapse; δ-catenin interacts directly with Shank3, which allows targeting of postsynaptic sites. Shank3 is an upstream node of the PI3K/AKT signaling pathway, and Rictor is a key regulatory and structural subunit of mTORC2 signaling. Downregulation of Rictor leads to altered expression of postsynaptic proteins such as GluR1 and ELKS; GSK-3β forms a complex with delta-catenin.

There are many lines of evidence suggest the association between *CTNND2* and autism spectrum disorder. *CTNND2* gene knockout mouse models exhibit behaviors characteristic of autism spectrum disorder and display a reduction in the density of dendritic spines in the hippocampus. The deletion of exon 2 of the *CTNND2* gene is linked to a number of neurological deficits, including social impairment in the prefrontal cortex of the brain, loss of dendritic spines, damage to inhibitory neurons, and the inhibition of the phosphatidylinositol-3-kinase (PI3K)/protein kinase B (Akt) signaling pathway. Melatonin enhances the synaptic function of gamma-aminobutyric neurons by triggering the PI3K/Akt signaling cascade, which provides a potential therapeutic target for neurodevelopmental disorders caused by *CTNND2* deficiency (Wang L. Y. et al., 2024). Figure 3 summarizes the currently available studies with a focus on CTNND2-related neuronal disorders and potential underlying mechanisms.

There are also many studies on the role of δ-catenin in cancer development and progression through a range of molecular actions, including Wnt/β-catenin signaling pathway. The expression of δ-catenin can be regulated by the repressive transcription factor Hes1 and activating transcription factor E2F1 during the progression of prostate cancer (Lu et al., 2010). δ-catenin may impede the invasion of medulloblastoma cells by inhibiting the EMT pathway, and has predicts a positive prognosis of medulloblastoma patients (Hu et al., 2022). δ-catenin signaling in neural diseases and cancer is summarized in Table 2.

**TABLE 2:** δ-catenin signaling in neural diseases and cancer and clinical implications.

Disease	Clinical relevance	Animal model	Biomarker value	Treatment strategy	References
Prostate cancer	In prostate cancer patients, δ-catenin expression level is increased	Increased expression of δ-catenin in a mouse model of transplanted NE-10 tumor with prostate neuroendocrine cancer	δ-catenin can form complexes with E− Cadherin, p120, α-catenin and β-catenin and participate in the Wnt signaling pathway	Combined treatment with anti-androgen and anti-β-catenin targets clinically significant prostate cancer with high levels of δ-catenin	Oh et al. (2023), Wang et al. (2009), Burger et al. (2002), Zhang et al. (2018b), Lu et al. (2010)
Liver cancer	δ-catenin expression is increased in hepatocellular carcinoma patients and δ-catenin enhances hepatocellular carcinoma progression	Knockdown of δ-catenin significantly reduced tumors in a subcutaneous hormonal tumor model in ICR mice	δ-catenin may be a target of HIF1α and miR-122–5p	Treatment of hepatocellular carcinoma by regulating the EIF3J-AS1 gene that plays a key role in liver cancer progression and is affected by *CTNND2*	Huang et al. (2019), Yang et al. (2019)
Lung cancer	δ-catenin expression was increased in patients with advanced cancer compared to the control group	When LLC cells were injected into the tail vein of B6/C57 mice, the *CTNND2*-KO group showed less lesions on the lungs and bones	δ-catenin synergistically enhances G1-S phase transition with classical Wnt signaling in Lewis lung cells	δ-catenin may become a new potential direct target for the treatment of lung adenocarcinoma	Huang et al. (2018), Xie et al. (2024)
Esophageal Carcinoma	SNP array copy number analysis showed that δ-catenin expression was increased in patients with esophageal cancer compared with the control group	The analysis of ESCC cell line rearrangement characteristics showed significant amplification of *CTNND2*	*CTNND2* is hypomethylate and increased in promoter expression in esophageal cancer	Focusing on the degree of promoter methylation of *CTNND2* may help prevent esophageal cancer	Singh et al. (2015), Maity et al. (2022), Brown et al. (2011), Singh et al. (2019)
Glioblastoma	The expression of δ-catenin increased in human glioma cells and promoted the invasiveness of glioma cells	δ-catenin was upregulated by bevacizumab treatment in human glioma cell xenotransplantation models	δ-catenin is upregulated in glioma cells and is associated with the transformation of the very aggressive mesenchymal phenotype of glioma cells	Combined with the synergistic ability of bevacizumab and δ-catenin, targeted therapeutic intervention was performed	Frattini et al. (2013), Shimizu et al. (2019)
Cri-du-Chat Syndrome	Mutations in *CTNND2* in patients with Cri-du-Chat Syndrome	Loss of δ-catenin in the delta-catenin N-term mice model or knocking out δ-catenin in pyramidal neurons damages the width and length of the spine head	Loss of δ−catenin during development alters synaptic structure, leading to developmental abnormalities	Early diagnosis based on the chromosome alteration characteristics of CNND2 and improved prognosis through early rehabilitation and educational intervention	Yuan et al. (2015), Sardina et al. (2014), Sheth et al. (2012), Cerruti Mainardi (2006)
Autism spectrum disorder	*CTNND2* is a neurodevelopmental gene that is downregulated in people with autism	*CTNND2* knockout mice showed decreased dendrite spine density	Loss of *CTNND2* is associated with inhibition of PI3K/Akt signaling	Melatonin therapy and MT therapy improve synaptic function and help improve autism spectrum disorders	Коваленко et al. (2020), Wang et al., 2024a; Xu et al. (2023b)
Myopia	Multiple SNPS of *CTNND2* gene were significantly associated with myopia	In guinea pig models, *CTNND2* is differentially regulated by FSK	The SNP polymorphism of *CTNND2* gene is genetically associated with the development of myopia	The SNP polymorphism of *CTNND2* gene is genetically associated with the development of myopia	Yu et al. (2012), Wen et al. (2023), Liu and Zhang (2014), Srinivasalu et al. (2018), Li et al., 2011a
Alzheimer’s disease	The SNPS of *CTNND2*, rs17183619, rs13155993 and rs13170756, are significantly associated with Alzheimer’s disease	In Tg2576 transgenic AD mouse models, a new AD risk factor locus was found on the *CTNND2* gene	In Tg2576 transgenic AD mouse models, a new AD risk factor locus was found on the *CTNND2* gene	*CTNND2* may serve as a biomarker for AD that could be used for early detection and longitudinal monitoring of this devastating neurodegenerative disease	Koutras and Lévesque (2011), Koutras et al. (2011), Jun et al. (2012), Moncaster et al. (2022)

## 5 Opportunities for treatment of δ-catenin-associated disorders

The aberrant expression or mutation of δ-catenin has been linked to a range of diseases, including cat-call syndrome (CdCS), autism spectrum disorder (ASD), and certain types of cancer. Most recent research advancements have shed light on therapeutic developments in the context of δ-catenin signaling.

Firstly, gene replacement therapy for cat-call syndrome (CdCS). CdCS is caused by heterozygous deletion of the short arm of chromosome 5 (5p15.2), and *CTNND2* deficiency and leads to mental retardation and synaptic developmental abnormalities. The gene replacement therapy restored CTNND2 expression and improves cognitive function in a rat model of CdCS constructed by CRISPR/Cas9 and successfully elevated δ-catenin levels in the brain, alleviated neuroinflammation and synaptic pathology, and significantly ameliorated cognitive deficits using an adeno-associated virus (AAVPHP.eB) vector (Shen et al., 2025).

Second, the development of small-molecule drugs targets neurodevelopmental disorders associated with *CTNND2* deletion. *CTNND2* knockout mouse models have demonstrated that its deletion affects synaptic protein synthesis through the downregulation of the PI3K/Akt/mTOR pathway, resulting in cerebellar developmental deficits and abnormalities in motor function. These findings provide a foundation for the potential development of PI3K/Akt/mTOR pathway activators or compounds that enhance synaptic plasticity (e.g., NMDA receptor modulators) (Wang L. et al., 2024). Conversely, the inflammatory response in the prefrontal cortex and hippocampus in the CdCS model suggests that combined anti-inflammatory agents (e.g., JAK/STAT inhibitors) may enhance efficacy (Shen et al., 2025).

Third, targeted therapy against *CTNND2* fusion gene-driven cancer. In non-small cell lung cancer, the *CTNND2-ROS1* fusion gene has been identified as a critical driver of tumor growth, operating through the activation of the ROS1 tyrosine kinase. This pathway is susceptible to ALK inhibitors, such as crizotinib and loratinib, highlighting a targeted therapeutic approach with potential efficacy enhancement. Therefore, the development of more efficient second-generation inhibitors (e.g., Repotinib, Taletrectinib) against CTNND2-ROS1 fusion is crucial to overcome the resistance mutation.

Fourth, the potential of CTNND2-based therapy for neurodegenerative diseases. δ-catenin plays a crucial role in synaptic stability and neuronal survival, and its aberrant expression has been linked to diseases such as Alzheimer’s disease (Koutras and Lévesque, 2011). A promising therapeutic strategy involves increasing δ-catenin expression through the use of small molecules that either activate *CTNND2* transcription or inhibit its degradation, such as ubiquitinase inhibitors. Alternatively, the use of histone deacetylase inhibitors in the context of epigenetic modifications has been explored as a means to upregulate δ-catenin expression.

## 6 Prospect and challenges

The field of *CTNND2*/δ-catenin research is rapidly developing, bringing many exciting discoveries, but also many challenges. Here are some key directions and potential difficulties for future research.

δ-catenin plays an important role in the development and function of the nervous system. However, the precise mechanisms of action remain unclear, and further researches are required to elucidate the exact mechanisms that δ-catenin influences neurodevelopment, especially in terms of synaptic formation and maturation. For instance, further investigation is required to ascertain how δ-catenin regulates synaptic maturation and neuronal excitability, whether δ-catenin is associated with other aspects of the nervous system, and how these processes are linked to the development of disorders, such as intellectual disability and autism spectrum disorder. In addition, cancer is often accompanied by dysregulation of neuro-regulation, and whether the role of δ-catenin in cancer pathogenesis is through neural regulation, such as regulating neurons or axonal progression.

Second, *CTNND2* gene variations. Alterations of *CTNND2* are associated with a range of conditions, including intellectual disability, autism, attention deficit hyperactivity disorder, and cancer. However, whether *CTNND2* can be exploited to serve as potential therapeutic target remains unclear. Further researches are required to determine the role of *CTNND2* variations in larger populations and their exact association with these diseases and to explore potential therapeutic targets. A better understanding of the function and mechanism on δ-catenin would help explore therapeutic strategies targeting *CTNND2* expression or function to treat the related diseases, *e.g.*, the development of small molecule drugs, gene therapies, or cell therapies.

Third, technical challenges on *CTNND2* researches. It is very difficult to precisely manipulate of δ-catenin expression and to simulate δ-catenin function in complex biological systems. Novel tools and models need to be developed to elucidate the function of δ-catenin *in vivo*, particularly focus on its role in the brain. *CTNND2* is one of the genes unique to humans that may have played a pivotal role in the evolution of the human brain. Further studies are required to elucidate the role of *CTNND2* in human evolution, particularly focus on its effects on the characteristics of cortical synapses, including prolonged maturation and neoteny of synapses. And all of those investigations require more advanced technology.

In summary, the field of *CTNND2* gene research is full of opportunities and challenges. With the advancement of science and technology and the innovation of research methods, we are expected to have a deeper understanding of *CTNND2* in the next few years and provide new strategies for the diagnosis and treatment of related diseases. However, in-depth studies are clearly warranted in the future to understand the definitive roles of *CTNND2/*δ-catenin signaling in physiology and pathology, *e.g.*, neural disorders and cancers, and to translate findings into clinical management of those diseases.
